# *Potentilla alba* Extracts Affect the Viability and Proliferation of Non-Cancerous and Cancerous Colon Human Epithelial Cells

**DOI:** 10.3390/molecules25133080

**Published:** 2020-07-06

**Authors:** Klaudia Kowalik, Roman Paduch, Jakub W. Strawa, Adrian Wiater, Kamila Wlizło, Adam Waśko, Iwona Wertel, Anna Pawłowska, Monika Tomczykowa, Michał Tomczyk

**Affiliations:** 1 Department of Biotechnology, Microbiology and Human Nutrition, University of Life Sciences in Lublin, ul. Skromna 8, 20-704 Lublin, Poland; klaudia.kowalik@up.lublin.pl (K.K.); adam.wasko@up.lublin.pl (A.W.); 2 Department of Virology and Immunology, Institute of Biological Sciences, Maria Curie-Skłodowska University, ul. Akademicka 19, 20-033 Lublin, Poland; rpaduch@poczta.umcs.lublin.pl; 3 Department of General Ophthalmology, Medical University, ul. Chmielna 1, 20-079 Lublin, Poland; 4 Department of Pharmacognosy, Faculty of Pharmacy, Medical University of Białystok, ul. Mickiewicza 2a, 15-230 Białystok, Poland; jakub.strawa@umb.edu.pl; 5 Department of Industrial and Environmental Microbiology, Institute of Biological Sciences, Maria Curie-Skłodowska University, ul. Akademicka 19, 20-033 Lublin, Poland; adrianw2@poczta.umcs.lublin.pl (A.W.); kamila.wlizlo@poczta.umcs.lublin.pl (K.W.); 6 Independent Laboratory of Cancer Diagnostics and Immunology, I Chair and Department of Oncological Gynaecology and Gynaecology, Medical University of Lublin, ul. Staszica 16, 20-081 Lublin, Poland; iwona.wertel@umlub.pl (I.W.); anna.pawlowska@umlub.pl (A.P.); 7 Department of Organic Chemistry, Faculty of Pharmacy, Medical University of Białystok, ul. Mickiewicza 2a, 15-222 Białystok, Poland; monika.tomczyk@umb.edu.pl

**Keywords:** *Potentilla alba*, colon cancer, HT-29, CCD 841 CoTr, chemoprevention, polyphenols, antioxidants, cytotoxic effect

## Abstract

The aim of this study was to determine the anti-tumor activity of extracts isolated from *Potentilla alba* L. on human colon cancer cells of the HT-29 line and on non-cancer colon epithelial cells of the CCD 841 CoTr line. The research methods we used to determine the cytotoxic and proliferative properties were 3-(4,5-dimethylthiazol-2-yl)-2,5-diphenyltetrazolium bromide (MTT) and neutral red (NR) assays, the ability to produce nitric oxide, the Griess method, and the biochemical properties like 2,2-diphenyl-1-picrylhydrazyl (DPPH) and ferric reducing antioxidant power (FRAP) methods indicating reduction activity of tested samples. In order to obtain a phytochemical profile of the different extracts an analytical method based on liquid chromatography-photodiode array detection-electrospray ionization ion-trap time-of-flight mass spectrometry (LC-PDA-ESI-MS/TOF) was applied. Finally, the effects of the extracts on the morphology and cell counts were assessed by May–Grünwald–Giemsa staining. After a comprehensive analysis of all the experiments, the extracts were found to demonstrate cytotoxic properties, they stimulated the division of non-cancer cells, and they were able to scavenge free radicals. In the NR method, the cell viability dropped to approximately 80% compared to the control. In the MTT assay, tumor cell proliferation decreased to 9.5% compared to the control. Therefore, we concluded that this plant has medical potential.

## 1. Introduction

Colorectal cancer (CRC) is an epithelial malignant tumor that develops in the colon or rectum. According to The Global Cancer Observatory (GCO), colorectal cancer is very common in the world and ranks second in terms of mortality [1]. Surgical treatments and chemotherapy are the most commonly used methods of CRC treatment. Scientists are searching for new treatments, as chemotherapy has many side effects and a large proportion of patients die due to the cancer’s recurrence [2]. Many previous studies have shown that colorectal cancer is sensitive to plant-derived substances [3,4]. Among them polyphenols are classified as very strong antioxidants that neutralize the toxic effects of selected radicals, which are responsible for DNA damage [5]. Reactive oxygen species cause oxidative stress. Their presence can lead to an increase in the proliferation rate, mutation in DNA, cell adaptation and genomic instability. As a consequence of these processes, cancer may develop.

According to previous studies, *Potentilla alba* Linn. (Rosaceae) is used in Ukrainian medicine to reduce the amount of thyroxine in the blood, which is important in thyroid disease. However, in traditional medicine, *P. alba* helps for problems with intestine secretions, and can be used to treat intestinal colic, diarrhea, and ulcerative colitis. Preparations from *P. alba* help to prevent dental caries, protect the mucosa from toxins and irritants, and heal wounds, burns, and infections [6,7]. The white cinquefoil possesses also anti-inflammatory, immuno-modulating, hepatoprotective, and adaptogenic properties which depends on secondary metabolites such as flavonoids, tannins, and triterpenes presented in this plant [8,9,10]. The aim of our study was to examine the antiproliferative, antioxidant, and anti-cancer activity of selected white cinquefoil extracts obtained solvents by different polarity on human non-cancer phenotype control and cancerous colon cells. 

## 2. Results

### 2.1. Chemical Characterization

In order to obtain a phytochemical profile of the different extracts and fractions, PAL1–PAL7 of *P*. *alba* were obtained from solvents with increasing polarity, and an analytical method based on liquid chromatography-photodiode array detection-electrospray ionization ion-trap time-of-flight mass spectrometry (LC-PDA-ESI-MS/TOF) was applied. The LC-MS profile showed the presence of a large group of polyphenolic compounds (33 compounds) corresponding to the protonated molecular ions of different types of flavonoids, such as quercetin, kaempferol, and isorhamnetin derivatives (**12–25, 29, 30**) and also caffeoylquinic acids (**1–4, 6, 7**) or procyanidins (**26–28, 32, 33**) (Table 1). In addition, flavan-3-ol derivatives (**5, 8–11, 31**) were confirmed. Individual polyphenolic compounds in PAL1–PAL7 samples were identified by comparison of their UV spectra and the m/z values were identified with those of the selected compounds described in the literature. 

In all cases, our results revealed that qualitative profiles of the PAL1–PAL7 extracts seemed to be very similar. The LC-MS spectra suggested that diglycosylated quercetin isomers (**13** and **14**) were flavonols that dominated in all analyzed samples. These compounds gave the presence of the [M + H]^+^ ion at m/z 597 and UV maximum at 265 and 350 nm. The analysis of the LC-MS spectra of compounds **13** and **14** suggested quercetin *O*-pentoso-hexoside isomers. In particular, by the analysis of mass spectrum of the [M + H]^+^ ion, compounds **13** and **14** showed product ions accounting for the same composition of quercetin *O*-xylosylo-hexoside, such as quercetin *O*-sambubioside. Our results also revealed that identified compounds **16** and **17** seemed to be very similar to **13** and **14** and suggested them to be potential kaempferol *O*-pentoso-hexoside isomers, such as kaempferol *O*-sambubioside. A large quantitative content of procyanidin derivative (**28**) in two analyzed extracts, PAL1 and PA7, was also detected. Another 10 interesting monomer, dimer, and trimer isomers were also detected in the same extracts. In the (Supplementary Materials, Figure SF1), a figure of the UV spectrum of the major constituents of the analyzed PAL1–PAL7 extracts are available.

### 2.2. Cytotoxicity

#### 2.2.1. Neutral Red (NR) Uptake Assay

**Figure 1:**
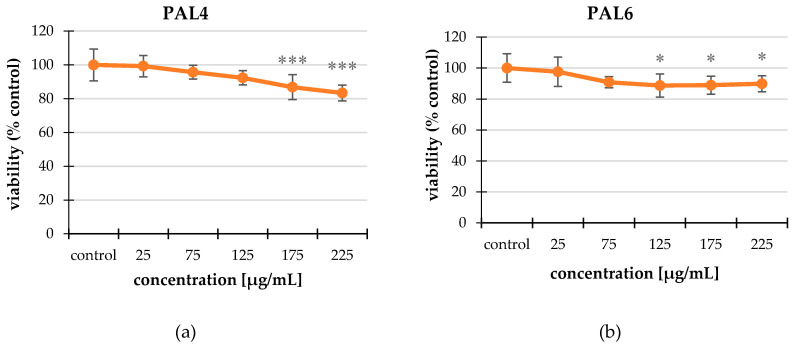

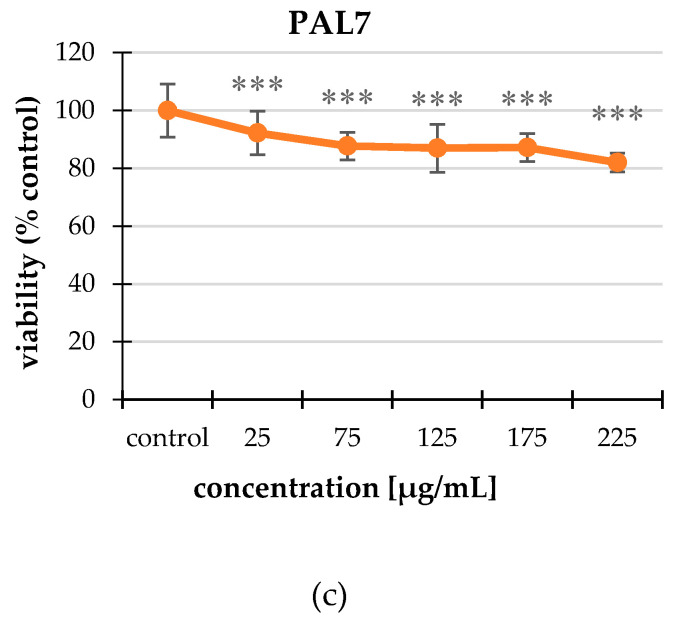
The effect of the extracts on the cell viability of the HT-29 line studied using a neutral red (NR) uptake assay. The most effective extracts were PAL4 (**a**), PAL6 (**b**), and PAL7 (**c**). The values are compared to the control, regarded as 100%; * *p* < 0.01, ** *p* < 0.005, *** *p* < 0.001, one-way ANOVA, Dunnett’s test.

In our study extracts concentrations 0–225 μg/mL were used. The cytotoxicity of the tested extracts was assessed in relation to the HT-29 (Figure 1) and CCD 841 CoTr lines (Supplementary Materials, Figure S2). All tested extracts showed cytotoxicity in a dose-dependent manner (Table 2 and Table 3).

PAL4 and PAL7 were the strongest in relation to both lines. The difference in the effect of the extracts in relation to the non-cancer and cancer lines was visible when PAL3 and PAL6 were used. PAL3 caused a decrease in viability up to 84.2% compared to the control; PAL6 caused a decrease in viability up to 89.88% compared to the control. This difference may be due to the different composition of the extracts. Quercetin *O*-pentoso-hexoside isomer was the most abundant in PAL3, and 5-*O*-p-coumaroylquinic acid was the most abundant in PAL6.

#### 2.2.2. MTT Assay

**Figure 2:**
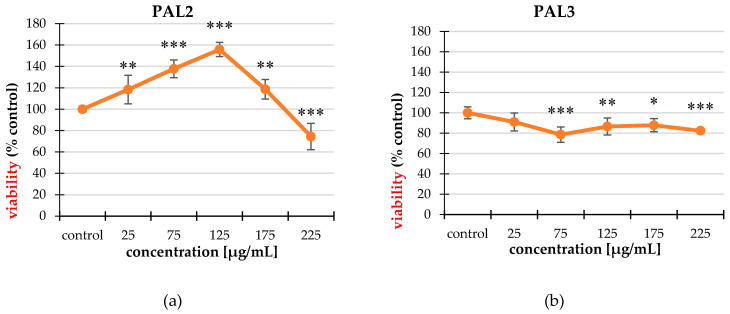

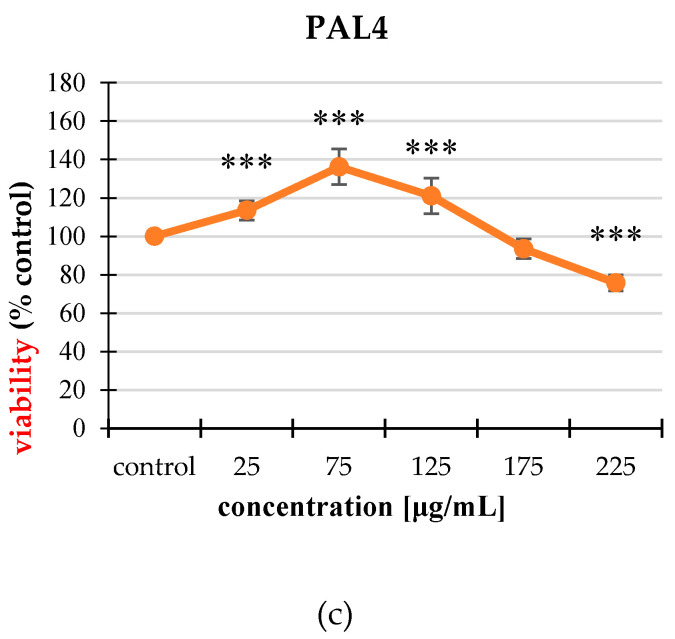
The cytotoxic properties of the tested extracts of the HT-29 line studied using an 3-(4,5-dimethylthiazol-2-yl)-2,5-diphenyltetrazolium bromide (MTT) assay. The most effective extracts were PAL2 (**a**), PAL3 (**b**), PAL4 (**c**). The values are compared to the control regarded as 100%; * *p* < 0.01, ** *p* < 0.005, *** *p* < 0.001, one-way ANOVA, Dunnett’s test.

In the 3-(4,5-dimethylthiazol-2-yl)-2,5-diphenyltetrazolium bromide (MTT) assay, the differences in cell viability are visible. The extracts did not cause statistically significant decreases in viability of the non-cancer phenotype control cell line, even in the highest concentrations used (225 μg/mL) (data not shown). In the case of the HT-29 line, the largest influences were from PAL2, PAL3, and PAL4 (Figure 2). PAL2 and PAL4 were the most cytotoxic at the highest concentration (225 μg/mL) while PAL3 was the most cytotoxic at the 75 µg/mL concentration. These differences may be due to the different functionality of non-cancer control and cancer cells.

### 2.3. Cell Proliferation

**Figure 3:**
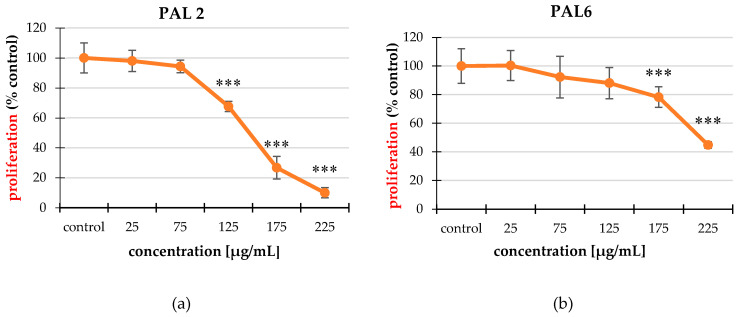

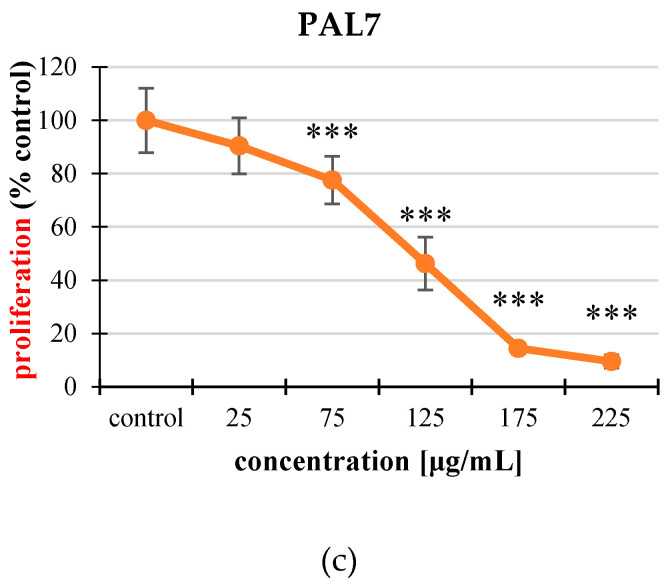
Inhibition of the line proliferation of the HT-29 line studied using an MTT assay. The most effective extracts were PAL2 (**a**), PAL6 (**b**), and PAL7 (**c**). The values are compared to the control regarded as 100%; * *p* < 0.01, ** *p* < 0.005, *** *p* < 0.001, one-way ANOVA, Dunnett’s test.

The tested extracts acted in different ways on the non-cancer and cancerous cell lines. For the HT-29 cancer cell line, all extracts significantly reduced the cell proliferation and they acted contrary to the CCD 841 CoTr line. PAL2, PAL6, and PAL7 were the strongest in both lines (Figure 3) and (Supplementary Materials, Figure S3). The effect was dose-dependent. The difference in the activity of the extracts was significant. In the case of the cancer line, the extracts caused a proliferation decrease up to 9.50% compared to the control, and, in the non-cancer phenotype control line, they caused an increase in the cellular division up to 239.78% compared to the control.

### 2.4. Cellular Morphology

Cell staining confirmed the observations obtained in the NR uptake and MTT assay. Cell morphology of both the non-cancer and cancer lines looked different after treatment with the extracts compared to the control. The most significant difference in cell morphology of HT29 and CCD 841 CoTr cells was the size of the cells. Intestinal epithelial cells were larger. In addition, tumor cells tended to group together and CCD 841 CoTr grew evenly dispersed across the culture surface. Moreover, the cytoplasm of tumor cells was significantly less expressed than in intestinal epithelial cells. CCD 841 CoTr cells before treatment expressed characteristic large cell nucleus and visible cytoplasm surrounding the nucleus. In addition, the cells tended to interact directly with each other. Cell staining was also intense. After these cells treatment with tested compounds they tended to take on a more elongated shape than was observed under control conditions. The cell nucleus was significantly reduced and cytoplasm contraction was found. The surface area of the observed cells was also smaller than under control conditions. This would suggest weaker cell adhesion to the surface due to their smaller volume. Differences in the morphology of treated and untreated cancer cells were: complete contraction of the cytoplasm, tendency to separate cells from each other, and also lost the characteristic star-like morphology pattern of treated cells. 

**Figure 4:**
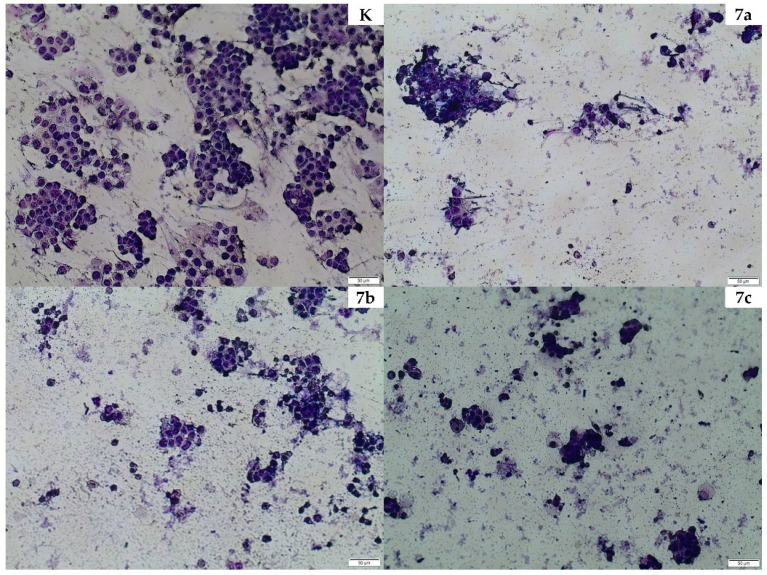
Presentation of the effect of PAL4 extract on morphology and cell count of the HT-29 line. Images were taken using an Olympus BX51 light microscope. K—control, 7a—25 μg/mL, 7b—75 μg/mL, and 7c—125 μg/mL.

A similar effect was observed in both cell lines regardless of the concentration of tested agent. These extracts expressed lower activity towards the non-cancer phenotype cell line. PAL4 turned out to be the strongest in relation to the HT-29 line. In the case of the CCD 841 CoTr line, PAL3 in all concentrations proved to be the strongest. The effect was dose-dependent. The images show that the extracts caused a reduction in the number of cells and influenced their morphology (Figure 4) and (Supplementary Materials, Figure S4).

### 2.5. Antioxidant Properties

**Figure 5:**
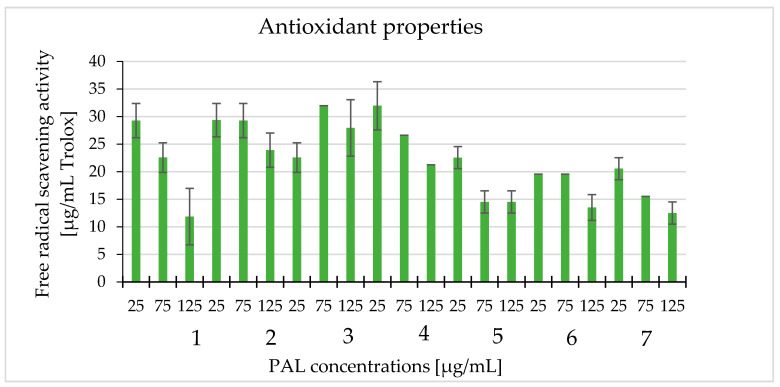
The antioxidant properties of the tested extracts (PAL1–PAL7) at the concentrations 25, 75, and 125 μg/mL with the 2,2-diphenyl-1-picrylhydrazyl (DPPH) radical scavenging method and expressed as Trolox equivalents (μg/mL).

The antioxidant capacity of white cinquefoil extracts was determined using a DPPH assay. Three concentrations of each extract were tested in the experiment: 25, 75, and 125 µg/mL. The results showed that all extracts had antioxidant activity. We also observed a downward trend in the antioxidant activity as the concentration increased. The highest DPPH radical reducing activity was observed when the 25 μg/mL concentration was used. The highest antioxidant capacity was recorded for PAL3 (27.93 µg/mL) and PAL4 (31.95 µg/mL), expressed as an equivalent of Trolox. PAL1, at the concentration of 125 µg/mL, had the lowest scavenging capacity for free radicals (11.84 µg/mL), expressed as an equivalent Trolox (Figure 5).

### 2.6. Ferric Reducing of Antioxidant Power Assay—FRAP Method (Fe^3+^ Ion Reduction)

**Figure 6:**
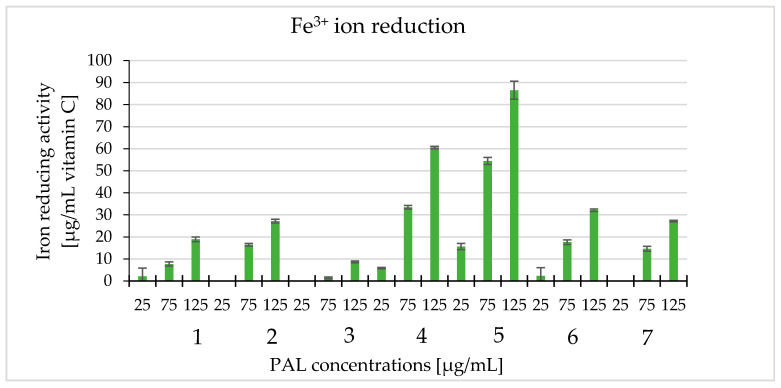
The ability to reduce iron ions of the tested extracts (PAL1–PAL7) at the concentrations 25, 75, and 125 μg/mL with the ferric reducing antioxidant power (FRAP) method and expressed as vitamin C equivalents (μg/mL).

We determined the ability of white cinquefoil extracts to reduce Fe^3+^ ions using the FRAP method. Extracts in the 25–125 µg/mL concentration range were used for the experiment. These extracts have the ability to reduce Fe^3+^ ions and it increased with higher extract concentrations. The highest value was recorded at the concentration of 125 µg/mL of PAL5 (86.5 µg/mL) as an equivalent of ascorbic acid. The lowest value was 1.40 µg/mL as an equivalent of vitamin C, which appeared when PAL3 was used at the concentration of 75 µg/mL (Figure 6). As can we see on Figure 6 at concentrations of 25 µg/mL for PAL2, PAL3, and PAL7 no activities were observed.

### 2.7. Chelating Iron Ions

**Figure 7:**
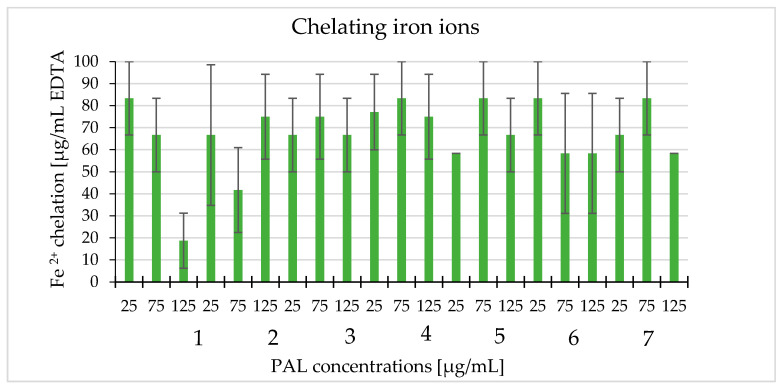
The ability of the tested extracts to chelate iron ions (PAL1–PAL7) at the concentrations 25, 75, and 125 μg/mL expressed as EDTA equivalents (μg/mL).

Using this method, we determined the ability of white cinquefoil extracts to chelate Fe^2+^. Extracts in the 25–125 µg/mL concentration range were tested and the results showed that they had the ability to chelate Fe^2+^ ions. There was no downward or upward trend in ion chelation depending on concentration. The highest level of chelation occurred with PAL1 and PAL6 at the concentration of 25 µg/mL and in PAL4, PAL5, and PAL7 at the concentration of 75 µg/mL (83.33 µg/mL), expressed as an equivalent of ethylenediaminetetraacetic (EDTA). PAL1 at a concentration of 125 µg/mL had the lowest ability to chelate Fe^2+^ ions (18.75 µg/mL) expressed as an equivalent of EDTA (Figure 7).

### 2.8. Nitric Oxide (NOx) Production

**Figure 8:**
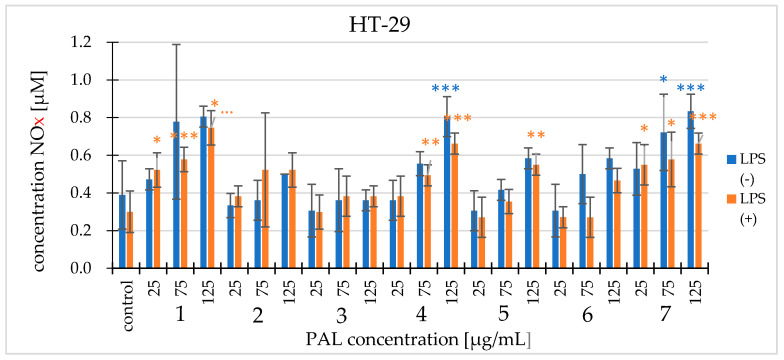
The effect of extracts on the production of nitric oxide by the HT-29 cell line. The nitric oxide levels were tested with the addition of lipopolysaccharide (LPS) and without LPS. The values are compared to the control regarded as 100%; * *p* < 0.01, ** *p* < 0.005, *** *p* <0.001, one-way ANOVA, Dunnett’s test.

The nitric oxide level was determined using two experimental variants: with and without cells pre-incubated with lipopolysaccharide (LPS). LPS is an endotoxin that occurs in the outer membrane of Gram-negative bacteria walls [11]. Extracts at concentrations of 25, 75, and 125 µg/mL were tested. For both lines, the concentration of nitric oxide produced was higher or at a similar level as in the control. In most cases, a higher NOx concentration occurred in experimental variants without cells preincubation with LPS. There are differences between the lines. In the cancer line, the concentration of nitric oxide increased with increasing concentration, whereas in the non-cancer phenotype line, the concentration could not be observed. PAL7 showed the strongest effect on the HT-29 cells (increased to 0.83 ± 0.09 µM NOx compared to the control (0.39 ± 0.18 µM)), and the most effective against CCD 841 CoTr cells was PAL5 (increase to 0.84 ± 0.16 µM in comparison to the control (0.47 ± 0.16 µM) (Figure 8) and (Supplementary Materials, Figure S5).

## 3. Discussion

In the present study, the activity of selected extracts PAL1-PAL3, PAL7, and fractions PAL4-PAL6 of white cinquefoil *P. alba* on HT-29 cancer cells and the transformed CCD 841 CoTr cell line were used to examine potential antiproliferative activities. Although the antiproliferative inhibitory properties using these both cell types such as CCD 841 CoN or colon epithelial features have been studied [12,13]. At the beginning of the study, the hypothesis was adopted that selected extracts of white cinquefoil had a different impact on cancerous as compared to non-cancer phenotype control cells of the human colon epithelium. After the research, we concluded that this hypothesis was supported.

There are many studies indicating the effect of various plant extracts on cells of different types of cancer, but often non-cancer cell lines are not used in the studies. In the present study, we wanted to check how these extracts and fractions act on non-cancer cells and whether they are toxic to them [14,15]. The observed effects of all tested PAL1-PAL7 can be explained by the presence of different types of polyphenolic compounds such as quercetin, kaempferol, procyanidins, and caffeoylquinic acids, since all compounds are the major phytoconstituents thereof. These polyphenolics can prevent the DNA damage caused by free radicals or carcinogenic agents through diverse mechanisms [16,17,18]. There is evidence that polyphenolic compounds have strong antioxidant effects [19,20].

In this study, LC-MS analysis was performed for the determination of the polyphenolic profiles of the PAL1-PAL7 for the identification of thirty-three polyphenolics, as summarized in Table 1. According to our experiments, all of these extracts contain quercetin *O*-pentoso-hexoside isomer which was found to be the most abundant ingredient and could be responsible for the most observed effects. Quercetin and its derivatives demonstrate strong anti-inflammatory properties that can be expressed on different cell types. It directly inhibited tumor necrosis factor (TNF-α) activation by c-Jun NH-terminal kinase (JNK), c-Jun, extracellular signal-related kinase (ERK), and nuclear factor κB (NF-κB). In contrast, quercetin indirectly increased c-receptor the peroxisome proliferator-activated receptor (PPARγ) activity, thereby antagonizing the transcriptional activation of inflammatory genes by NF-κB or protein-1 activator (AP-1) [21,22]. Similar, kaempferol affects many intracellular and extracellular mechanisms involved in cell signaling that are responsible for cancer progression. Studies have shown that this flavonols works on a wide range of cancers, including colorectal cancer [18,22]. Han Jin Cho and Jung Han Yoon Park [19] explained the possible mechanism of action of kaempferol on colon cancer. It can cause G1 or G2/M arrest. In the first case, it affected the reduced expression of cyclin D1, E, A, CDK2, and 4, the phosphorylation of Rb, and G1 arrest. In the second case, it affected the expression of Cdc25C, cyclin B1, and Cdc2, which results in a decrease of Cdc2 activity and G2/M arrest [19]. Procyanidins are another important polyphenolic compound present in analyzed samples. They affect many different processes in the body, e.g., the cell cycle (reduction expression of p53, c-myc, and cyclin D1, E, A, and B), arrest in the G1 phase, apoptosis signaling (reduction expression of Bcl-2, activation of Cas-9 and Cas-3), affect transcription factors and kinases (reduction expression of the mitogen-activated protein kinase (MAPK) protein and MAPK protein phosphorylation). They also cause reduction of matrix metallopeptidase (MMP) 9 and angiogenic vascular endothelial growth factor (VEGF) expression, migration capability, tumor cell proliferation, and increase apoptosis [23].

Recent studies conducted on the HT-29 line showed that caffeoylquinic acids were absorbed and metabolized by colon cancer cells and reduced cell viability suggesting that they modulate cell cycle processes with increased apoptosis [24]. Although polyphenols are well-known as potent antioxidants, the pro-oxidative effect is associated with their apoptotic effect in various types of cancer cells. It should be noted, however, that proxidative compounds increase ROS cell levels to cytotoxic levels in cancer cells, but not in non-cancer phenotype cells. This effect can be explained by a higher concentration of copper ions and greater metabolic activity in cancer cells compared to non-cancer phenotype control cells. The hypothesis is confirmed by the results of biochemical tests, such as the FRAP, DDPH, or iron chelating methods [25,26].

Our study confirmed results obtained by other authors [27,28]. They also showed that white cinquefoil has a large amount of polyphenolics, such as procyanidins and flavonoid derivatives. It has antioxidant properties and was able to protect oxidizable substrates from HO-mediated degradation. After reviewing the literature, there were few publications describing the study of white cinquefoil extracts on non-cancer and cancer cell lines. There were works showing the effects of other *Potentilla* species on some cancers. It was shown that the polysaccharide isolated from *P. anserina* has immunomodulatory activity in vivo and in vitro. It affected lactate dehydrogenase (LDH), interferon gamma (IFN-γ), interleukin 10 (IL-10), acyl carrier protein (ACP), and lymphocyte function, suggesting its subsequent therapeutic use [29].

Another study by Paduch and co-workers suggested that analyzed extracts obtained from different species of the genus *Potentilla* possessed strong antioxidant and immunomodulatory properties reducing the level of IL-10 and increasing the level of IL-6 suggesting that these plants could be used to prevent disturbances in the functioning of the large intestine [30]. Another experiment carried out by Hyun-Ju Yu and co-authors showed that the extract of *P. discolor* caused a significant increase in the apoptosis of human mucoepidermoid carcinoma cells by increasing the expression of proapoptotic p53 upregulated modulator of apoptosis (PUMA) protein and reducing the phosphorylation signal transducers and activators of transcription [31]. An important finding was also on the *P. chinensis* species and its impact in osteosarcoma cancer cells. The study showed that *P. chinensis* has anti-cancer properties with a reduced effect on the non-cancer control line, suggesting the use of this plant as a potential therapeutic agent [32]. In addition, *P. fulgens* studies have shown cytotoxicity to various tumor lines: A-549 lung, THP-1 leukemia, OVCAR-5 ovary, SF-295 neuroblastoma, HEP-2 liver, and PC-3 prostate. They also showed high antioxidant capacity [33]. The extract of this plant also induced apoptosis in relation to the MCF-7 and U87 lines and reduction of the endogenous glutathione level [34]. *P. reptans* and its effect on the mouse breast cancer cell line have also been studied. Unfortunately, this species showed low levels of cytotoxicity [35].

Previous biological studies on various species from the Rosaceae family inspired us to study *P. alba*, as a strong therapeutic chemopreventive potential. In our present experiment, the NR uptake assay was used to assess the cytotoxic activity on HT-29 tumor cells and non-cancer CCD 841 CoTr. After analyzing the results, we concluded that the extracts of white cinquefoil were cytotoxic to the above-mentioned cell lines. It can be presumed that the extracts from this plant affected the integrity of the cell membranes, damaging them. In the HT-29 line, the cell viability dropped to 82%. All the extracts influenced this line except PAL3. The viability of non-cancer control cells also decreased to 77%; however, in this case PAL6 expressed much less activity. This suggested that this extract was an effective cytotoxic compound, because at higher concentrations, it affected the viability of the cancer cells and had no effect on non-cancer control colon cells. These results were confirmed by May–Grünwald–Giemsa staining, which illustrated changes in the cell numbers.

Another method for assessing the cytotoxic activity was the MTT assay. In the case of this method, PAL2, PAL3, and PAL4 acted on the tumor line. In contrast to non-cancer control cells, the extracts were not cytotoxic and did not cause a decrease in the viability in the context of cellular metabolism. After analyzing the results obtained from this study, we concluded that the extracts of the white cinquefoil had little effect on the mitochondrial metabolism of cancer cells. The results from both cytotoxicity studies did not coincide, which means that extracts from *P. alba* acted on the integrity of cell membranes and, to a lesser extent, interfered with the cell metabolism. The lack of influence on non-cancer phenotype cells demonstrated in the MTT test may be justified in their metabolism. Cells of the HT-29 line directed at growth and survival, and obtaining energy in the process of glycolysis may redirect the mitochondrial potential to the synthesis of biomolecules to meet the demand [36]. Overexpression of enzyme and transporter synthesis also confirms this trend [37].

The obtained results indicated that the tested extracts significantly affected the proliferation of both the HT-29 and CCD 841 CoTr cell lines. In the case of cancer lines, these extracts reduced the proliferation by up to 10%. In the case of CCD 841 CoTr cells, these extracts significantly increased the level of proliferation, even several times. The obtained results showed that these extracts had a much better effect on cell multiplication than on their elimination. This suggests that they need a longer time to act on the cells and likely affected one of the stages of the cell cycle by blocking it on the HT-29 line or stimulating it on the CCD 841 CoTr cells. The observed differences in proliferative activity may also result not only from the activity of the examined factors but also the speed of doubling time (DT) of the population of tested cells. For cancer ones it was about 30 h and for CCD 841 CoTr line about 40 h.

Tumor cells have uncontrolled cell divisions that are not under internal control, which leads to uninhibited proliferation. That is why it is so important to stop their reproduction. It can be assumed that the cytotoxicity and influence on cell proliferation were caused by the content of phenolic compounds. The results of our research suggested the use of the tested extracts in lower concentrations, to encourage action on the tumor cells and to affect the non-cancer control cells to a lesser extent. The extracts examined in the presented work also showed the ability to scavenge free radicals, chelate Fe^2+^ ions, and reduce Fe^3+^ ions. As is known from the literature, reactive oxygen species under the conditions of homeostasis play the role of regulators and mediators in many processes occurring in the cell. An imbalance causes reactive oxygen species to take part in the initiation and activation of many harmful processes. They cause the destruction of cell components: nucleic acids, proteins, and lipids. An excessive and sustained increase in free radical production is involved in the pathogenesis of cancer, atherosclerosis, diabetes, and neurodegenerative diseases.

To maintain balance, the body has developed a so-called antioxidant system [38]. Studies on the chemical composition of the genus *Potentilla* have shown that the genus contains a large amount of polyphenols. Therefore, we can conclude that the high content of these compounds affects the ability of the white cinquefoil to scavenge free radicals. This is due to the fact that it is mainly polyphenols that are responsible for antioxidant activity [8]. Scientific data shows that nitric oxide is a free radical messenger that regulates various biological functions in the body. In cells, it can work in two ways; NO may have pro- and anti-tumor activity or high levels of NO may have anti-cancer effects.

At high concentrations, NO produces a cytotoxic effect and promotes apoptosis [39]. In the tests carried out in the presented work, the analysis of nitric oxide level was performed using the Griess method. After a specified time, both the control cells and those that were incubated with the extracts generated NO. In most cases compared to the control, both the HT-29 line and CCD 841 CoTr cells increased the nitric oxide levels of extracts PAL1, PAL4, and PAL7 in both lines and extract 5 in the non-cancer cell line. This may suggest that high NO concentrations are cytotoxic and is confirmed by the NR method.

In summary, after a comprehensive analysis of our all experiments, the extracts and fractions of *P. alba* demonstrated cytotoxic properties, stimulated the proliferation of non-cancer control cells, and were able to scavenge free radicals. The analyzed white cinquefoil extracts led to changes in the integrity of the cell membranes of the HT-29 and CCD 841 CoTr cells and caused small changes in the mitochondrial metabolism. These changes depended on the type of extract and cell line used. White cinquefoil extracts affected the proliferation of HT-29 and CCD 841 CoTr cells. The proliferation of HT-29 cells was significantly reduced, while the proliferation of non-cancer control cells was significantly stimulated by extracts. The paper assumes the comparison of non-cancer vs. cancer cells. However, the result may also be affected by the use of embryonic vs. mature cells. The reactivity of cells of varying degrees of maturity could also have an effect on the final result obtained. However, the full answer to this question requires further research aimed only at determining the differences in cell reactivity to test compounds based on their maturity. Extracts showed also the ability to reduce Fe^3+^ ions, chelate Fe^2+^ ions, and scavenge free radicals. *P. alba* extracts reduced the number of and caused morphological changes to HT-29 and CCD 841 CoTr cells. The induction or inhibition of nitric oxide production depended on the concentration of the extract and the cell line used. Therefore, we concluded that this plant may be considered as a helpful compound in phytotherapy; however, further research should be carried out.

## 4. Materials and Methods

### 4.1. Materials

#### 4.1.1. Chemicals and Reagents

Acetonitrile Optima was purchased from Fisher Chemical (Thermo Fisher Scientific, Leicestershire, UK), and ultra-pure water (resistivity of 18.2 MΩ-cm) was obtained using the POLWATER DL3-100 system (Labopol, Kraków, Poland). Formic acid (Ph. Eur., Merck, Darmstadt, Germany) was used as the mobile phase modifier. (+)-catechin, (-)-epicatechin, and isorhamnetin were purchased from ROTH (Karlsruhe, Germany). Quercetin 3-*O*-glucoside, quercetin 3-*O*-rutinoside, kaempferol 3-*O*-glucoside, and kaempferol (purity > 96%) were isolated from flowers of *Ficaria verna* Hud. [40]. Kaempferol 3-*O*-galactoside (purity > 96%) was isolated from flowers of *Cirsium rivulare* (Jacq.) [41].

Quercetin and tiliroside (both purity > 96%) were isolated from the aerial parts of *Drymocallis rupestris* (L.) Soják [42]. Roswell Park Memorial Institute, culture medium (RPMI) 1640, Dulbecco, trypsin solution (0.25%) with the addition of 0.53 mM ethylenediaminetetraacetic acid (EDTA), dimethyl sulfoxide (DMSO), neutral red, lipopolysaccharide (LPS) *Escherichia coli*, sodium nitrite (NaNO_2_), p-aminobenzenesulfonamide, sodium dodecyl sulfate (SDS), 3-(4,5-dimethylthiazol -2-yl)-2,5-diphenyltetrazole bromide (MTT), Giemsa dye, sodium edetate (EDTA), ferrozine, sodium orthophosphate (Na_3_PO_4_) were from Sigma-Aldrich (Steinchein, Germany). Buffered saline (PBS) (with and without calcium and magnesium ions) was from Biomed, Lublin. Fetal bovine serum (FCS), penicillin, streptomycin, antibiotic-antimycotic (penicillin, streptomycin, and amphotericin B) were from Gibco. NaCl solution (0.85%) and deionized and distilled H_2_O were from the Department of Virology and Immunology UMCS in Lublin. 2,2-diphenyl-1-picrylhydrazil (DPPH) was from Sigma-Aldrich Co. (St. Louis, MO, USA). Formalin, calcium chloride (CaCl_2_), acetic acid, ethanol, methanol, N-(1-naphthyl) ethyleneamine, hydrochloric acid (HCl), May–Grünwald’s reagent, iron (II) chloride (FeCl_2_), ascorbic acid, acid trichloroacetic (TCA), potassium ferrocyanide, orthophosphoric acid (H_3_PO_4_), and iron (III) chloride (FeCl_3_) were from POCH (Gliwice, Poland).

#### 4.1.2. Plant Materials

*P*. *alba* seeds (index seminum—354) were gently obtained from the Hortus Botanicus University of Tartu, Estonia. Aerial parts of *Potentilla alba* L. (Rosaceae) were collected from plants in the garden of medicinal plants of Medical University of Białystok, Poland in July 2016–2018. The plant material was dried under shade and air ventilation at room temperature. M. Tomczyk confirmed the taxonomic identification. A voucher specimen (PAL-17039) was deposited in the Herbarium of the Department of Pharmacognosy (Medical University of Białystok, Poland).

### 4.2. Methods

#### 4.2.1. Cell Cultures

The studies were conducted on two cell lines: HT-29 (ATCC^®^ HTB-38™), a human colon cancer cell line, and CCD 841 CoTr (ATCC^®^ CRL-1807™), a human non-cancerous colon epithelial cells line. The HT-29 cells were cultured in RPMI 1640 medium with 10% or 2% FBS. In contrast, the CCD 841 CoTr cells were cultured in mixed media RPMI 1640 and Dulbecco (DMEM) (1:1) with 10% or 2% addition of FBS. Both lines were cultured in media supplemented with antibiotics (100 U/mL penicillin and 100 μg/mL streptomycin). The cultures were kept under standard conditions, i.e., temperature of 37 °C (HT-29), 34 °C (CCD 841 CoTr), and 5% CO_2_.

#### 4.2.2. Preparation of the Extracts

Powdered raw plant material (2.0 g each time) was extracted using an ultrasonic bath (Sonic-5, Polsonic, Poland) at a controlled temperature (40 ± 2 °C) for 45 min in a 1:100 (*w:v*) solvent ratio to obtain the PAL1 (methanol), PAL2 (50% methanol), and PAL3 (water) extracts. The filtered supernatants were reduced to dryness under a vacuum (Büchi System, Switzerland) at a controlled temperature (40 ± 2 °C) and further suspended in water and freeze-dried using a vacuum concentrator Labconco (Kansas City, MO, USA) until they reached a constant weight. Further, the powdered raw plant material (2.0 g) was then extracted with methanol (3 × 50 mL) or with 50 mL of 80% (*v:v*) methanol using an ultrasonic bath (Sonic-5, Polsonic, Warsaw, Poland) at a controlled temperature (40 ± 2 °C) for 45 min.

After solvent evaporation, these methanol extracts were diluted with water (50 mL) and partitioned using: chloroform (10 × 20 mL), diethyl ether (PAL4) (20 × 20 mL), ethyl acetate (PAL5) (20 × 20 mL), and n-butanol (PAL6) (20 × 20 mL). Finally, 2.0 g of the aerial parts of *P. alba* was extracted using an ultrasonic bath (Sonic-5, Polsonic,) at the same conditions to obtain PAL7 (80% acetone). All the concentrated fractions (PAL1–PAL7) were separately suspended in water and lyophilised using the vacuum concentrator (Labconco) until a constant weight. With this procedure, the observed yield values were 85 mg (PAL1), 113 mg (PAL2), 79 mg (PAL3), 21 mg (PAL4), 42 mg (PAL5), 74 mg (PAL6), and 99 mg (PAL7).

#### 4.2.3. LC-ESI-MS Analysis

The assessments of the chemical composition of each extract (PAL1–PAL7) were carried out on a 1260 Infinity LC (Agilent, Santa Clara, CA, USA) consisting of a binary pump, a column oven and photo-diode array (PDA) detector over a 55 min period. Mass Hunter software was used for instrument control and data acquisition during the analysis. The separation was performed using a Kinetex C18 column (150 × 3 mm, 2.6 µm) (Phenomenex, Torrance, CA USA). The mobile phase was 0.1% formic acid in water (A) and 0.1% formic acid in acetonitrile (B). The separation was achieved by a gradient of 0–1.5 min 5% B; 1.5–22 min 5%–28% B; 22–35 min 28%–75% B; 35–45 min 75%–95% B; 45–48 min 95% B; 48–49 min 95%–5% B; 49–55 min 5% B. The flow rate was 0.5 mL/min and the column temperature was maintained at 25 ± 0.8 °C. The UV–vis spectra was recorded from 190 to 540 nm with selective wavelength monitoring at 280 nm. MS detection was carried out on a 6230 LC/TOF (Agilent, Santa Clara, CA USA) mass spectrometer equipped with an electrospray ionization source with Agilent Jet Stream thermal focusing (AJS-ESI). The parameters used for the ionization source were set as follows: drying and sheath gas flow: 12 L/min; nebulizer: 35 psi; source temperature 350 °C; ion spray voltage 4500 V for the positive mode analysis. The data were collected in the 115–1900 m/z range and processing was performed using the Mass Hunter Qualitative analysis software.

#### 4.2.4. Samples Preparation

The prepared lyophilizates were weighed and dissolved in the appropriate volume of culture liquid RPMI 1640 and DMSO (1:1). The final solution concentration was 100 mg/mL. The final DMSO concentration at the highest concentration of samples used did not exceed 0.25%. Based on our previous studies, this is not a concentration that would have a toxic effect in the assumed time range of experiments.

#### 4.2.5. Cytotoxicity

##### Neutral Red (NR) Uptake Assay

The NR method is commonly used to determine the cytotoxicity of tested compounds. Neutral red (NR) is absorbed by living cells and bound in lysosomes. This dye is washed away from dead cells. In an acidified environment, the dye is released from the cells. The color intensity is directly proportional to the amount of dye captured and thus the number of viable cells [43]. The assay was performed on 96-well plates (100 µL 1 × 10^5^ cells/mL). After 24 hrs incubation with examined extracts or without them, the culture media was removed and 100 µL NR (40 µg/mL) was added. Incubation was performed for 3 hrs at 37 °C. After this time, the dye was removed and all wells were rinsed with 200 µL of 0.5% formalin in 1% CaCl_2_. After 2 min, the solution was removed and 100 µL of 1% glacial acetic acid in 50% ethanol was added to all wells. Thereafter, we performed extraction for 20 min with shaking at room temperature. Then, a spectrophotometric measurement of absorbance was done at a wavelength λ = 550 nm using an E_max_ precision microplate leader (Molecular Devices) spectrophotometric reader.

##### MTT Assay

This method was used to determine the cell viability and proliferation. The principle of the method is to measure the oxidoreductive activity of mitochondria using the MTT dye, which, in living cells, is reduced by succinate dehydrogenase to purple formazan crystals. The reaction is accompanied by a change in color from yellow to purple [44]. The assay was performed on 96-well plates (100 µL 1 × 10^5^ cells/mL). After 24 hrs incubation with examined extracts or without them, 25 μL of freshly prepared MTT solution (5 mg/mL) in 0.85% NaCl was introduced into each well. After a 3 hrs incubation, 100 μL of 10% SDS solution in 0.01 N HCl was added to all wells to stop the reaction and dissolve the resulting formazan crystals. Incubation for 24 hrs was performed, after which the absorbance at λ = 570 nm was determined spectrophotometrically using the EL800 Universal Microplate Reader-Bio-Tek Instruments spectrophotometric reader. The amount of formazan was directly proportional to the number of viable cells.

#### 4.2.6. Cell Proliferation Analysis

The proliferation assay was performed using the MTT test as above. It differed only in the number of cells (100 µL 1 × 10^5^ cells/mL) and the time of incubation of the cells with extracts. The experiments were performed for 96 hrs.

#### 4.2.7. Cellular Morphology Analysis

##### May–Grünwald–Giemsa Staining

This method was used to illustrate the changes that occurred in the cell morphology under the influence of the tested substances. The staining was performed on 24-well plates (1 mL 1 × 10^5^ cells/mL). After 24 hrs incubation with the examined extracts or without them, the culture media was removed and the cells were stained with 1 mL of May–Grünwald dye for 3 min at room temperature. Then, 1 mL of deionized water was added to each plate. After 3 min of incubation at room temperature, the liquid was removed. All plates were rinsed with deionized water, and then the cells were stained with Giemsa dye (dilution 1:20) for 30 min at room temperature. After this time, the dye was removed and the wells were rinsed with 1 mL of deionized water and then allowed to dry. The images were taken using an Olympus BX51 light microscope.

#### 4.2.8. Antioxidant Properties

##### DPPH Method

The DPPH method allowed testing of the antioxidant properties of a given compound or preparation. It is based on determining the degree of reduction of the stable radical, DPPH. Antioxidants reduce DPPH molecules by attaching a hydrogen atom to a nitrogen atom with an unpaired electron. As a result of this reaction, hydrazine is formed, which is accompanied by a change in the maximum absorbance from λ = 515 nm to λ = 405 nm, thereby changing the color from purple to yellow [45]. The assay was carried out in plastic 96-well plates. Dilutions of tested substances and Trolox in methanol were prepared. Trolox was the positive control and the standard. The negative control was methanol. Then, 100 mL dilutions of test substances, Trolox, and methanol were added to the wells. Then 100 μL of a previously prepared solution of DPPH (solution of DPPH in methanol at a concentration of 0.2 mg/mL) was added to all wells. After 10 min of incubation at room temperature, the absorbance was determined spectrophotometrically at λ = 515 nm using an E_max_ precision microplate leader Molecular Devices spectrophotometric reader.

#### 4.2.9. Ability to Reduce Iron Ions

##### FRAP Method

Dilutions of tested substances and vitamin C in deionized water were prepared to a final volume of 1 mL. Vitamin C was the positive control and the standard. Deionized water was a negative control. After this, 1 mL of trichlorohydric acid (TCA) was added and centrifuged at 2000× *g* for 10 min. We transferred 2 mL of supernatant to new tubes and 2 mL of deionized water and 0.5 mL of 0.1% ferric (III) chloride were added. Then, 100 µL of each tube was transferred to a 96-well plate and the spectrophotometric absorbance was measured at λ = 700 nm using the EL800 Universal Microplate Reader-Bio-Tek Instruments spectrophotometric reader.

#### 4.2.10. The Ability to Chelate Fe^2+^ Ions

Under normal conditions, Fe^2+^ ions are stored and transported by specific proteins (e.g., transferrin). Due to this, there is no reaction between the free iron ions and reactive oxygen species. Iron participates are engaged in the Fenton reaction, where they generate the formation of a hydroxyl radical. In contrast, radicals easily bind to lipids and cause their peroxidation. This can be the cause of many different diseases. It is important to determine which compounds have the ability to form complexes with metal ions using sigma bonds.

Many studies indicated that chelating agents have antioxidant properties because they limit the redox potential and thus stabilize the oxidized form of metal. Dilutions of the tested substances and EDTA in deionized water were prepared to a final volume of 500 μL. EDTA was the positive control and the standard. Deionized water was a negative control. Then, 1.85 mL of methanol, 50 μL of 2mM FeCl_2_, and 100 μL of ferrosine were added to each tube to initiate the reaction. The contents of each tube were mixed and incubated for 10 min at room temperature. Then, 100 µL of each tube was transferred to a 96-well plate and the spectrophotometric absorbance was measured at wavelength λ = 562 nm using the EL800 Universal Microplate Reader-Bio-Tek Instruments spectrophotometric reader.

#### 4.2.11. Determination of Nitric Oxide Level

##### Griess Test

The assay was performed on 24-well plates (1 mL 1 × 10^5^ cells/mL). After a 24 hrs period of incubation, 10 µL of lipopolysaccharide (LPS) from *Escherichia coli* was added to individual plates (the final concentration in each well was 10 µg/mL). The cells were preincubated for 2 hrs under standard conditions. After this time, the media was removed from all plates (both those with and without LPS). Then, the media, with or without extracts, was added and the plates were incubated for 24 hrs. To make a standard curve, a series of dilutions of the standard and sodium nitrite (NaNO_2_) at concentrations of 0, 1, and 5 µM were prepared. After the plates were incubated, the supernatant was removed from each well into plastic tubes and frozen at −80 °C. After thawing, previously prepared standards and thawed supernatants (100 µL/well) were applied to the wells of the 96-well plate. We added 100 µL of the previously prepared Griess reagent (1% sulfanilamide and 0.1% N-(1-naphthyl) ethylenediamine dihydrochloride in orthophosphoric acid) to each well and incubated for 10 min at room temperature. After the incubation, a spectrophotometric measurement was made at a wavelength of λ = 570 nm, using the EL800 Universal Microplate Reader-Bio-Tek Instruments spectrophotometric reader.

### 4.3. Statistical Analysis

The results of the experiments were developed in MS Excel 2013. Statistical data analysis was performed using STATISTICA 13.3 using a one-way ANOVA test and post-hoc Dunnett’s test (all columns compared to the control).

## Figures and Tables

**Table 1:** Liquid chromatography-photodiode array detection-electrospray ionization ion-trap time-of-flight mass spectrometry LC-PDA/MS-TOF identification of the major constituents of the analyzed PAL1–PAL7 extracts.

No.	*t_r_* (*min*)	Tentatively Identified Compounds	UV Maximum (*nm*)	[M + H]^+/^[M − H]^−^ (*m/z*)
1	7.64	3-*O*-caffeoylquinic acid	240(sh), 323	377/353, 191
2	9.60	3-*O*-p-coumaroylquinic acid	312	361, 303, 217, 144/337, 163
3	9.91	5-*O*-p-coumaroylquinic acid	312	361, 303, 217, 144/337, 190
4	10.46	5-*O*-caffeoylquinic acid *	295(sh), 325	377/707, 353, 191
5	10.60	catechin *	280	291/289
6	11.16	3-*O*-feruloylquinic acid	295(sh), 325	-/367, 193
7	11.47	4-*O*-caffeoylquinic acid	245, 295(sh), 325	377, 355/707, 353
8	12.22	(epi)catechin dimer isomer	278	579/577, 287
9	13.40	epicatechin *	280	291/289
10	14.40	(*epi*)catechin trimer isomer	285, 330	865/863, 287
11	14.70	(*epi*)catechin trimer isomer	280	865/863
12	15.17	quercetin derivatives	260, 350	565, 303/563, 741
13	15.94	quercetin-*O*-pentoso-hexoside isomer	255, 355	303, 465, 597/595
14	16.30	quercetin-*O*-pentoso-hexoside isomer	255, 355	303, 465, 597/595
15	17.45	quercetin 3-*O*-glucoside *	255, 355	303, 465/463
16	17.55	kaempferol-*O*-hexoso-hexoside	265, 350	278, 449, 603/-
17	17.64	kaempferol-*O*-pentoso-hexoside	265, 350	287, 449, 581/579
18	17.77	quercetin-*O*-hexoside	255, 355	303, 465/300, 463
19	17.84	isorhamnetin-*O*-pentoso-hexoside	255, 350	317, 479, 611, 633/-
20	18.10	quercetin 3-*O*-rutinoside *	255, 350	-/300, 463, 609
21	18.88	kaempferol 3-*O*-galactoside *	265, 350	287, 449/447
22	19.53	kaempferol 3-*O*-glucoside *	265, 350	287, 449/447
23	19.98	isorhamnetin *O*-hexoside	265, 355	317, 479/-
24	25.25	quercetin *	255, 370	301/303
25	25.51	tiliroside *	265, 310	595/593
26	25.67	procyanidin derivatives	267, 330	625/623
27	26.15	procyanidin derivatives	265, 315	595/593
28	27.05	procyanidin derivatives	223, 295	584/582
29	27.94	kaempferol *	265, 365	287/285
30	28.23	isorhamnetin *	260, 375	317/315
31	43.98	(*epi*)catechin dimer	275, 325, 410	609/-
32	44.71	procyanidin derivatives	275, 325, 410	593/-
33	45.39	procyanidin derivatives	330, 410	593/-

* reference substance.

**Table 2:** The effect of the PAL4, PAL6, and PAL7 on the cell viability of the HT-29 line studied using a neutral red (NR) uptake assay. The extracts worked in a dose-dependent manner. The values are compared to the control, regarded as 100%; * *p* < 0.01, ** *p* < 0.005, *** *p* < 0.001, one-way ANOVA, Dunnett’s test.

Extract	Concentration [μg/mL]	Viability (% Control)
**PAL4**	25	99.28 ± 6.31
	75	95.70 ± 4.10
	125	92.35 ± 4.21
	175	86.86 ± 7.43 ***
	225	83.39 ± 4.68 ***
**PAL6**	25	97.63 ± 9.51
	75	90.85 ± 3.62
	125	88.70 ± 7.42 *
	175	88.91 ± 5.80 *
	225	89.88 ± 5.17 *
**PAL7**	25	92.25 ± 7.47 ***
	75	87.73 ± 4.75 ***
	125	86.98 ± 8.29 ***
	175	87.19 ± 4.89 ***
	225	82.02 ± 3.18 ***

**Table 3:** The effect of the PAL3, PAL4, and PAL7 on the cell viability of the CCC 841 CoTr line studied using a neutral red (NR) uptake assay. The extracts worked in a dose-dependent manner. The values are compared to the control, regarded as 100%; * *p* < 0.01, ** *p* < 0.005, *** *p* < 0.001, one-way ANOVA, Dunnett’s test.

Extract	Concentration [μg/mL]	Viability (% Control)
**PAL3**	25	95.35 ± 4.58
	75	89.67 ± 5.25 ***
	125	89.82 ± 6.12 ***
	175	84.58 ± 6.69 ***
	225	84.21 ± 4.21 ***
**PAL4**	25	85.39 ± 3.84 ***
	75	79.26 ± 7.23 ***
	125	77.34 ± 7.18 ***
	175	80.89 ± 5.90 ***
	225	88.49 ± 7.87 ***
**PAL7**	25	96.25 ± 5.29
	75	90.25 ± 7.31 *
	125	87.11 ± 9.11 *
	175	85.74 ± 5.32 ***
	225	84.79 ± 3.60 ***

## Supplementary Materials

The supplementary materials are available online. Figure S1: UV spectrum of major constituents of analyzed PAL1-PAL7 extracts, Figure S2: The effect of the extracts on the cell viability of the CCD 841 CoTr line studied using a neutral red (NR) uptake assay. The most effective extracts were PAL3 (d), PAL4 (e), and PAL7 (f). The values are compared to the control, regarded as 100%; * *p* < 0.01, ** *p* < 0.005, *** *p* < 0.001, one-way ANOVA, Dunnett’s test, Figure S3: Stimulated proliferation of the CCD 841 CoTr line studied using an MTT assay. The most effective extracts were PAL2 (d), PAL6 (e), and PAL7 (f). The values are compared to the control regarded as 100%; * *p* < 0.01, ** *p* < 0.005, *** *p* < 0.001, one-way ANOVA, Dunnett’s test, Figure S4: Morphology and cell count after application of PAL3 extract on CCD 841 CoTr line. Images were taken using an Olympus BX51 light microscope. K—control, 7a—25 μg/mL, 7b—75 μg/mL, and 7c—125 μg/mL, Figure S5: The effect of extracts on the production of nitric oxide by the CCD 841 CoTr cell line. The nitric oxide levels were tested with the addition of lipopolysaccharide (LPS) and without LPS. The values are compared to the control regarded as 100%; * *p* < 0.01, ** *p* < 0.005, *** *p* <0.001, one-way ANOVA, Dunnett’s test.
