# Supplementary material for: Potentilla alba Extracts Affect the Viability and Proliferation of Non-Cancerous and Cancerous Colon Human Epithelial Cells
Source: Molecules. 2020 Jul 6;25(13):3080. doi: 10.3390/molecules25133080 (PMC7411782; doi:10.3390/molecules25133080)
Supplement: Supplementary file 1 [file molecules-25-03080-s001.pdf]

molecules

MDPI

Supplementary Materials

Potentilla alba Extracts Affect the Viability and Proliferation of Non-Cancerous and Cancerous Colon Human Epithelial Cells

Klaudia Kowalik [1], Roman Paduch [2,3], Jakub W. Strawa [4], Adrian Wiater [5] Kamila Wlizło [5], Adam Waśko [1], Iwona Wertel [6], Anna Pawłowska [6], Monika Tomczykowa [7] and Michał Tomczyk [4,*]

[1] Department of Biotechnology, Microbiology and Human Nutrition, University of Life Sciences in Lublin, ul. Skromna 8, 20-704 Lublin, Poland; klaudia.kowalik@up.lublin.pl (K.K.); adam.wasko@up.lublin.pl (A.Wa.)
[2] Department of Virology and Immunology, Institute of Biological Sciences, Maria Curie-Skłodowska University, ul. Akademicka 19, 20-033 Lublin, Poland; rpaduch@poczta.umcs.lublin.pl (R.P.)
[3] Department of General Ophthalmology, Medical University, ul. Chmielna 1, 20-079 Lublin, Poland; rpaduch@poczta.umcs.lublin.pl (R.P.)
[4] Department of Pharmacognosy, Faculty of Pharmacy, Medical University of Białystok, ul. Mickiewicza 2a, 15-230 Białystok, Poland; jakub.strawa@umb.edu.pl (J.S.); michal.tomczyk@umb.edu.pl (Mi.T.)
[5] Department of Industrial and Environmental Microbiology, Institute of Biological Sciences, Maria Curie-Skłodowska University, ul. Akademicka 19, 20-033 Lublin, Poland; adrianw2@poczta.umcs.lublin.pl (A.Wi.); kamila.wlizlo@poczta.umcs.lublin.pl (K.W.)
[6] Independent Laboratory of Cancer Diagnostics and Immunology, I Chair and Department of Oncological Gynaecology and Gynaecology, Medical University of Lublin, ul. Staszica 16, Lublin 20-081, Poland; iwona.wertel@umlub.pl (I.W.)
[7] Department of Organic Chemistry, Faculty of Pharmacy, Medical University of Białystok, ul. Mickiewicza 2a, 15-222 Białystok, Poland; monika.tomczyk@umb.edu.pl (Mo.T.)
* Correspondence: michal.tomczyk@umb.edu.pl (M.T.); Tel.: +48-85-748-5694

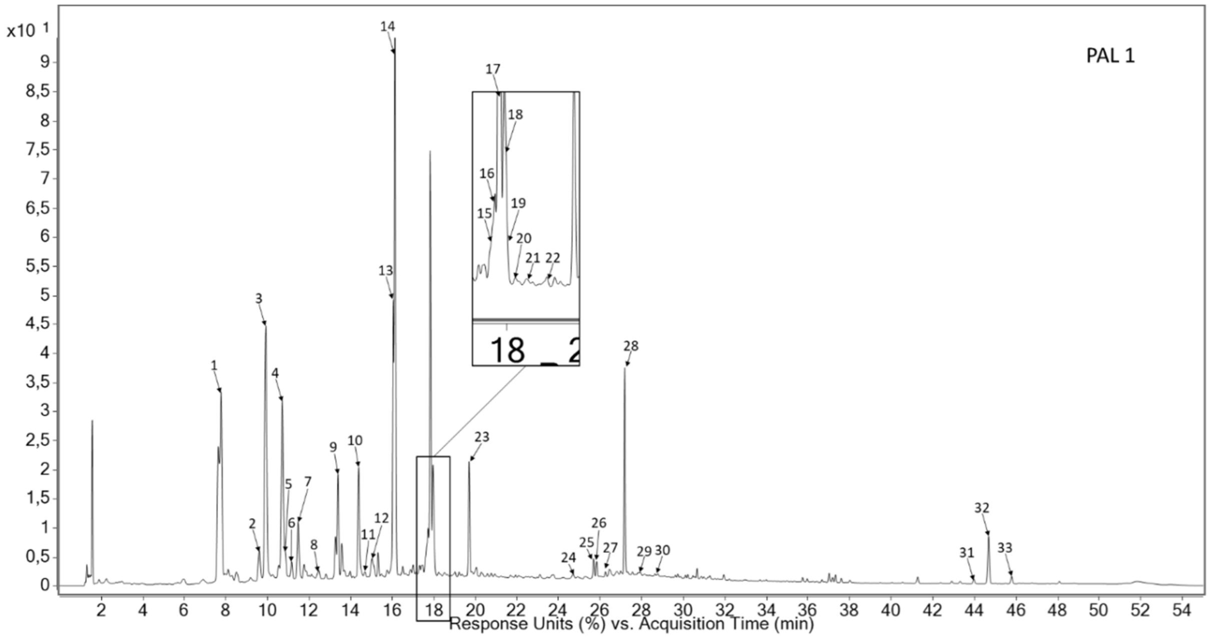

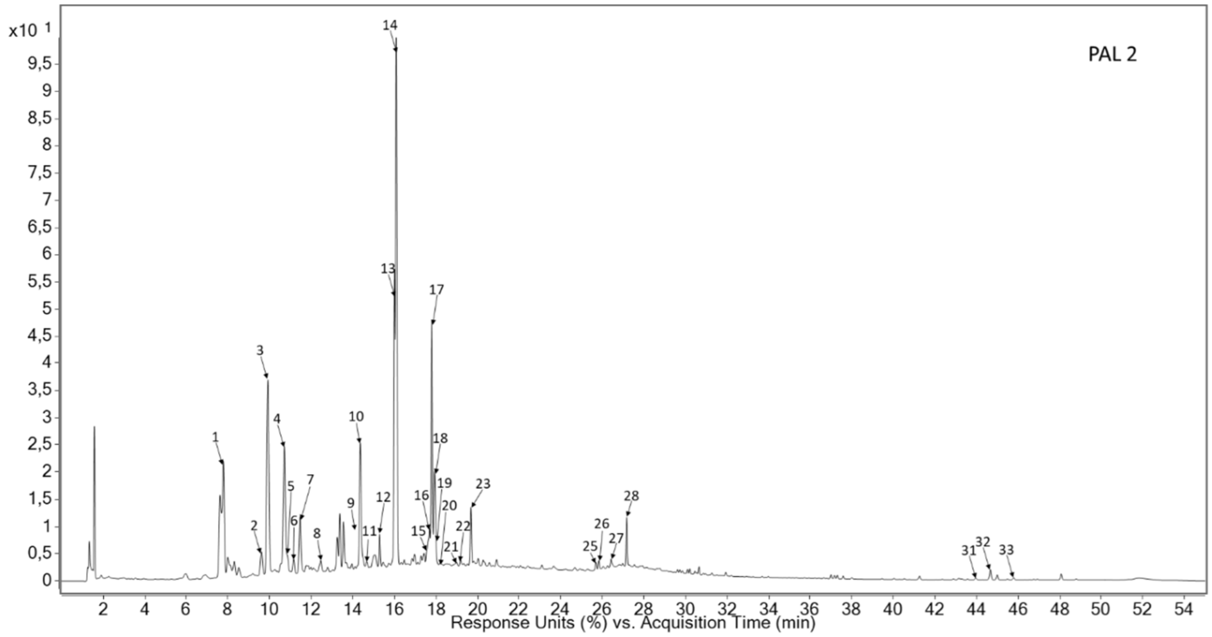
PAL 2
x10 1
Response Units (%) vs. Acquisition Time (min)

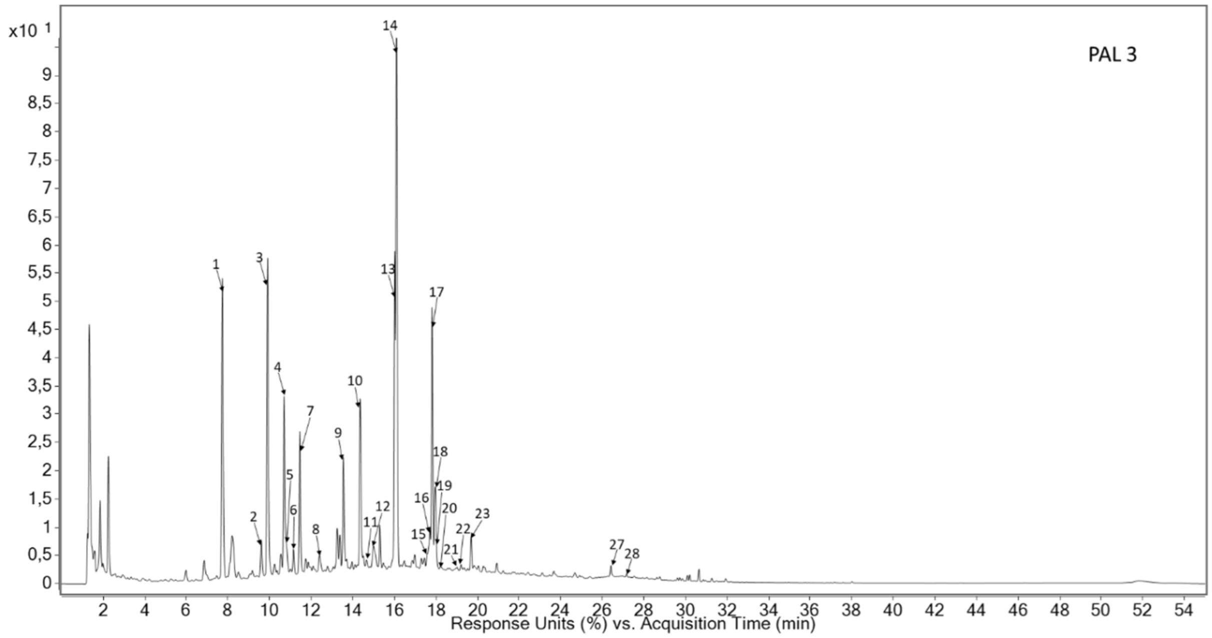
PAL 3
x10 1
Response Units (%) vs. Acquisition Time (min)

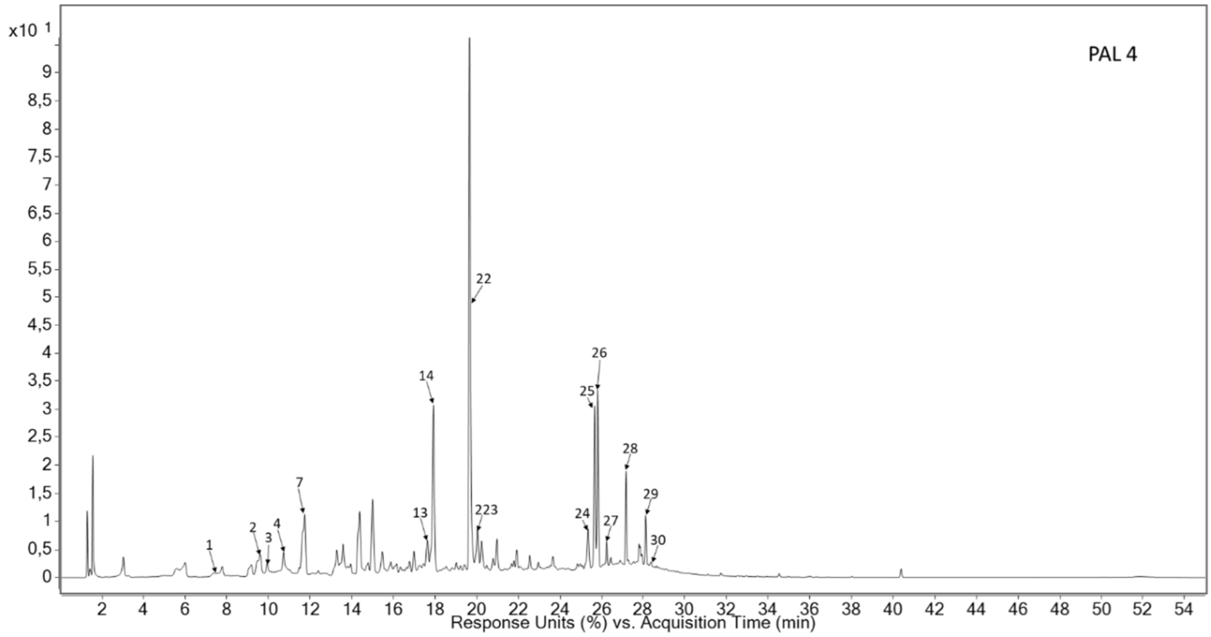
PAL 4
x10 1
Response Units (%) vs. Acquisition Time (min)

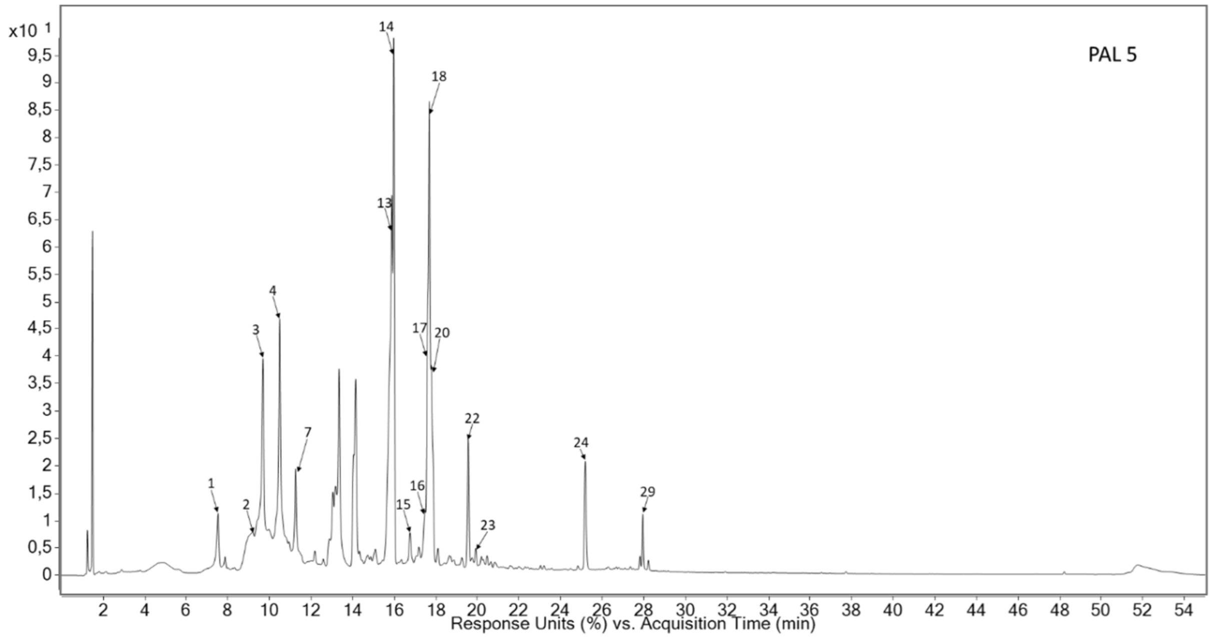
PAL 5
x10 1
Response Units (%) vs. Acquisition Time (min)

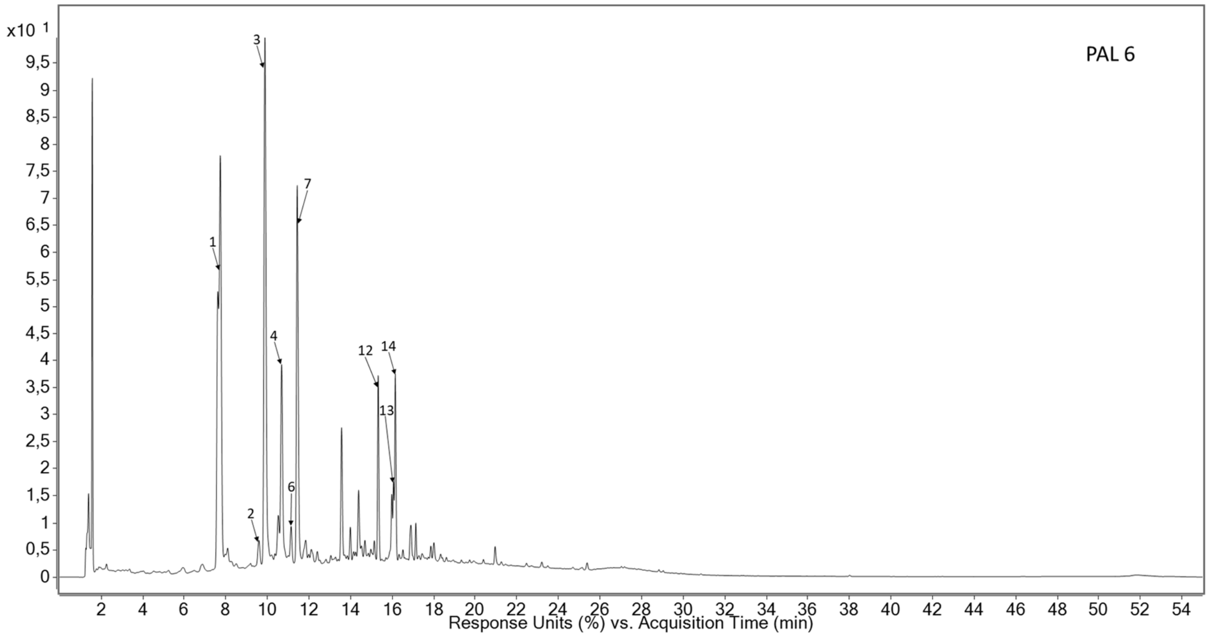

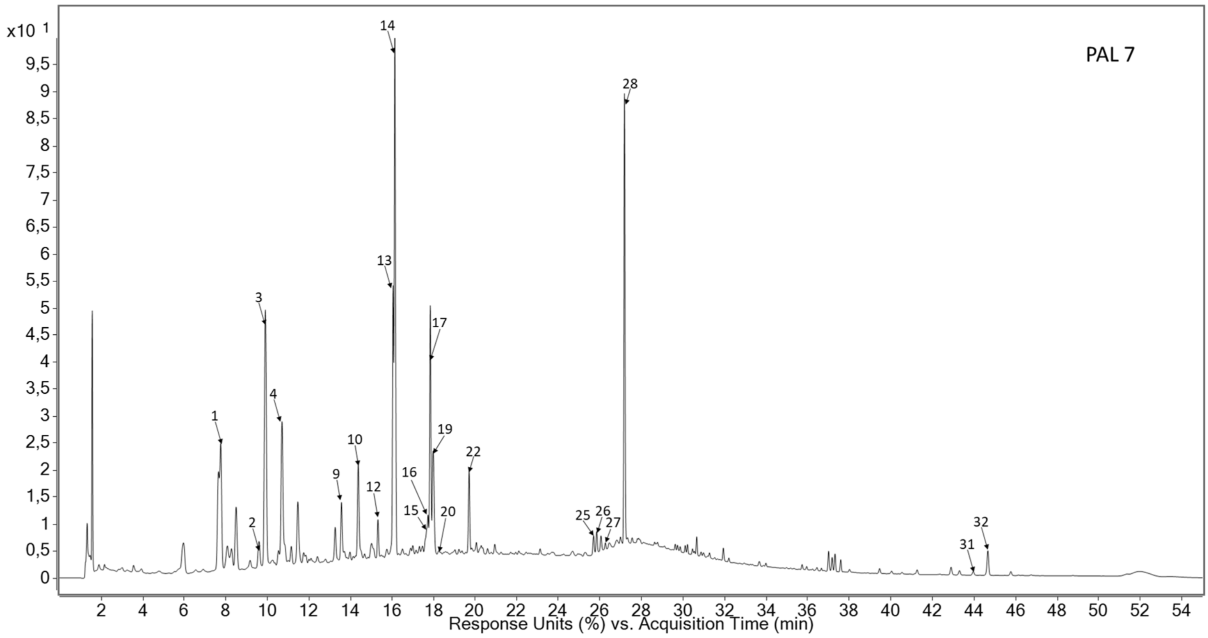

S1. UV spectrum of major constituents of analyzed PAL1-PAL7 extracts.

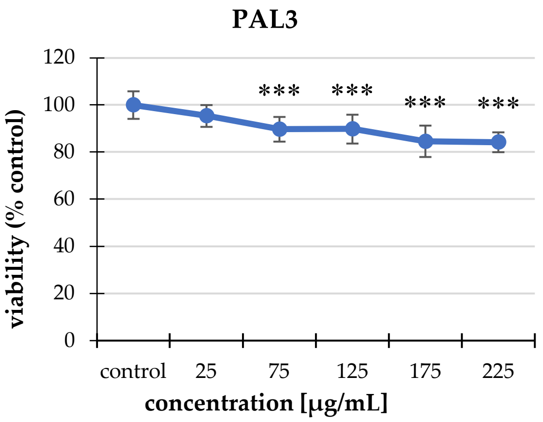

(d)

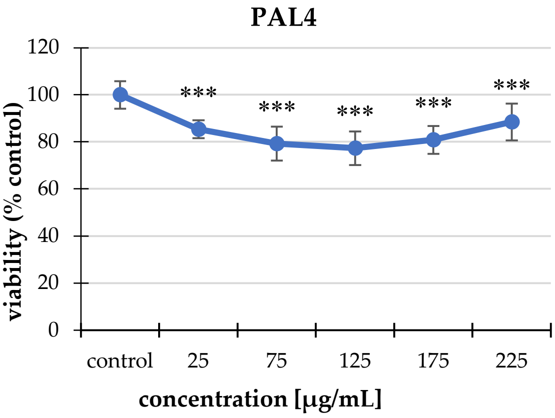

(e)

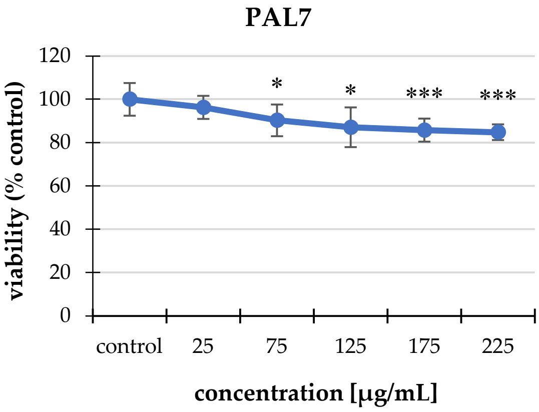

(f)

S2. The effect of the extracts on the cell viability of the CCD 841 CoTr line studied using a neutral red (NR) uptake assay. The most effective extracts were PAL3 (d), PAL4 (e), and PAL7 (f). The values are compared to the control, regarded as 100%; **p* < 0.01, ***p* < 0.005, ****p* < 0.001, one-way ANOVA, Dunnett's test.

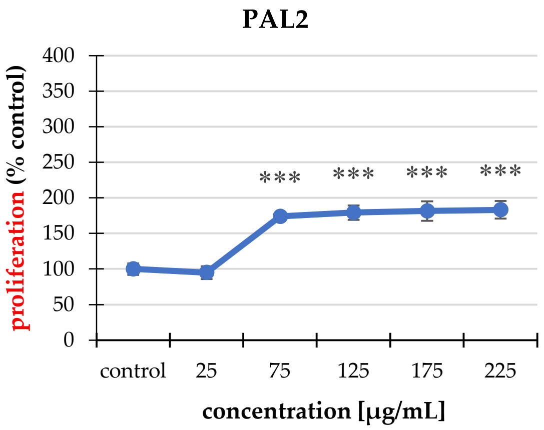

(d)

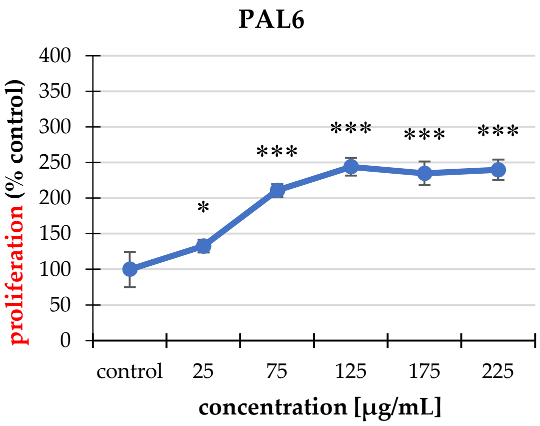

(e)

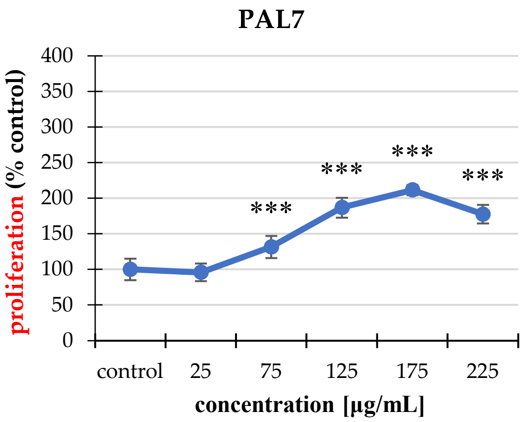

(f)

S3. Stimulated proliferation of the CCD 841 CoTr line studied using an MTT assay. The most effective extracts were PAL2 (d), PAL6 (e), and PAL7 (f). The values are compared to the control regarded as 100%; *$p < 0.01$, **$p < 0.005$, ***$p < 0.001$, one-way ANOVA, Dunnett's test.

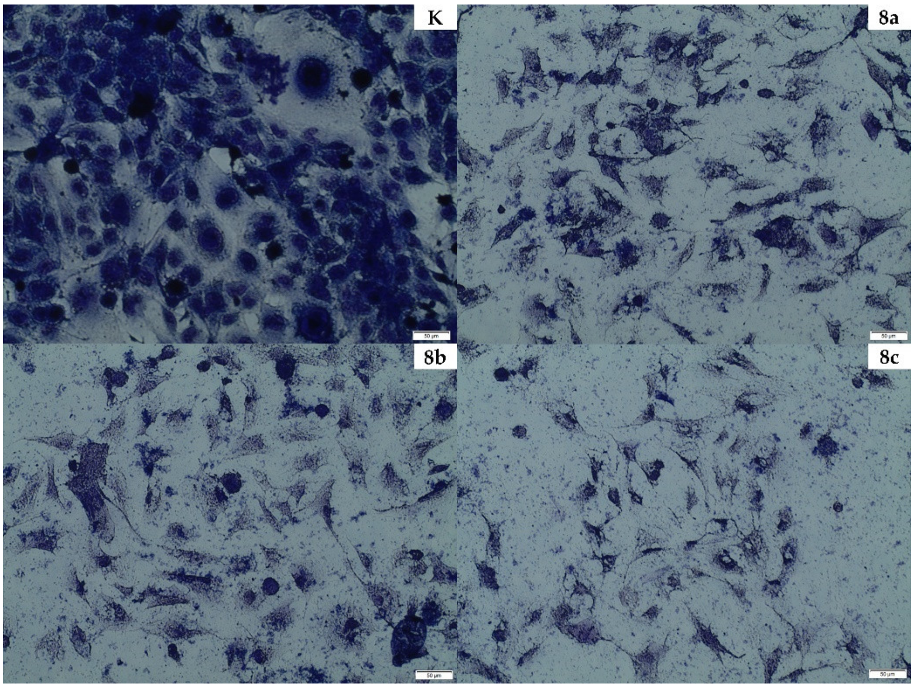

S4. Morphology and cell count after application of PAL3 extract on CCD 841 CoTr line. Images were taken using an Olympus BX51 light microscope. K—control, 7a—25 µg/mL, 7b—75 µg/mL, and 7c—125 µg/mL.

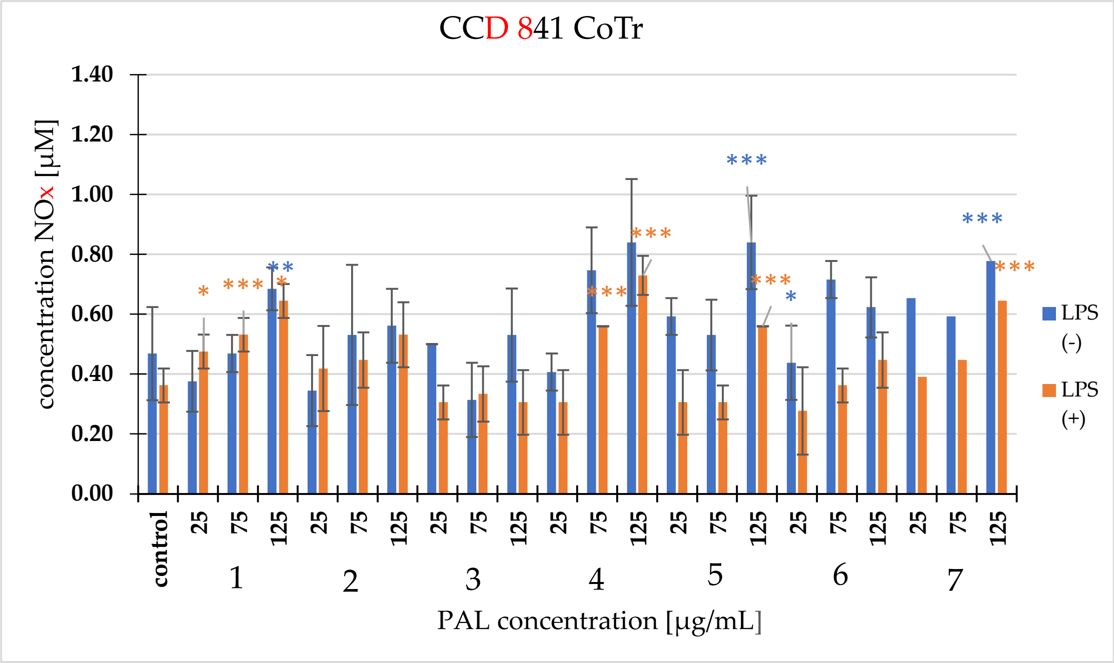

S5. The effect of extracts on the production of nitric oxide by the CCD 841 CoTr cell line. The nitric oxide levels were tested with the addition of lipopolysaccharide (LPS) and without LPS. The values are compared to the control regarded as 100%; *$p < 0.01$, **$p < 0.005$, *** $p < 0.001$, one-way ANOVA, Dunnett's test.
